# Activation of somatostatin 2 receptors in the brain and the periphery induces opposite changes in circulating ghrelin levels: functional implications

**DOI:** 10.3389/fendo.2012.00178

**Published:** 2013-01-11

**Authors:** Andreas Stengel, Yvette Taché

**Affiliations:** ^1^Division Psychosomatic Medicine and Psychotherapy, Department of Medicine, Obesity Center Berlin, Charité, Universitätsmedizin BerlinBerlin, Germany; ^2^Digestive Diseases Division, CURE: Digestive Diseases Research Center, Center for Neurobiology of Stress and Women’s Health, Department of Medicine, VA Greater Los Angeles Health Care System, University of California at Los AngelesLos Angeles, CA, USA

**Keywords:** X/A-like cell, somatostatin receptor subtypes, ghrelin cell, somatostatin receptor agonists and antagonist, cortistatin

## Abstract

Somatostatin is an important modulator of neurotransmission in the central nervous system and acts as a potent inhibitor of hormone and exocrine secretion and regulator of cell proliferation in the periphery. These pleiotropic actions occur through interaction with five G protein-coupled somatostatin receptor subtypes (sst_1__-__5_) that are widely expressed in the brain and peripheral organs. The characterization of somatostatin’s effects can be investigated by pharmacological or genetic approaches using newly developed selective sst agonists and antagonists and mice lacking specific sst subtypes. Recent evidence points toward a divergent action of somatostatin in the brain and in the periphery to regulate circulating levels of ghrelin, an orexigenic hormone produced by the endocrine X/A-like cells in the rat gastric mucosa. Somatostatin interacts with the sst_2_ in the brain to induce an increase in basal ghrelin plasma levels and counteracts the visceral stress-related decrease in circulating ghrelin. By contrast, stimulation of peripheral somatostatin-sst_2_ signaling results in the inhibition of basal ghrelin release and mediates the postoperative decrease in circulating ghrelin. The peripheral sst_2_-mediated reduction of plasma ghrelin is likely to involve a paracrine action of D cell-derived somatostatin acting on sst_2_ bearing X/A-like ghrelin cells in the gastric mucosa. The other member of the somatostatin family, named cortistatin, in addition to binding to sst_1__-__5_ also directly interacts with the ghrelin receptor and therefore may simultaneously modulate ghrelin release and actions at target sites bearing ghrelin receptors representing a link between the ghrelin and somatostatin systems.

## INTRODUCTION

In 1972, Guillemin’s group – while searching for additional releasing factors in hypothalamic extracts after their identification of thyrotropin-releasing hormone (TRH) – identified a novel negative regulator of pituitary somatotropic cells releasing growth hormone (GH; Brazeau et al., 1973). The cyclo peptide was named somatostatin (somatotropin release-inhibiting factor, SRIF), in keeping with its hypophysiotropic action (Guillemin, 2011). Somatostatin was found to be expressed in two biologically active isoforms: the tetradecapeptide somatostatin-14 (Brazeau et al., 1973) and the amino terminally extended octacosapeptide somatostatin-28 generated by differential post-translational processing from a common precursor molecule (Pradayrol et al., 1980). Thereafter, a flow of articles in rodents and humans established the ubiquitous distribution of somatostatin in various brain areas (Finley et al., 1981; Johansson et al., 1984; Uhl et al., 1985) and peripheral organs including the gastrointestinal tract (Costa et al., 1977; Walsh, 1994). This was followed by the identification and characterization of five distinct, high-affinity, specific somatostatin receptors (sst) encoded by five distinct genes (Gahete et al., 2010a). Structurally, these receptors belong to the so-called “superfamily” of membrane G protein-coupled (GPC) receptors. The sst_1-5_ have distinct as well as overlapping patterns of distribution in the brain (Fehlmann et al., 2000; Spary et al., 2008) and gut (Schafer and Meyerhof, 1999; Ludvigsen et al., 2004; Corleto et al., 2006) with a prominent expression of sst_2_ in the gastrointestinal tract (Sternini et al., 1997). Studies using new pharmacological tools, namely selective sst subtype agonists and antagonists (Grace et al., 2006; Cescato et al., 2008; Erchegyi et al., 2008, 2009; **Table 1**) point toward the role of different sst_1-5_ in mediating the large spectrum of somatostatin biological actions, mostly inhibitory in nature. Multiple effects of somatostatin can also result from the ability of sst to form both homodimers or heterodimers (sst_5_ with sst_1_ or dopamine D_2_ receptor, sst_2a_ with sst_3_, D_2_ or μ opioid receptor subtype 1) resulting in the activation of different intracellular signaling cascades (Rocheville et al., 2000; Baragli et al., 2007; Siehler et al., 2008).

**Table 1:** Structure and receptor binding affinity of somatostatin and somatostatin receptor agonists.

Peptide	Structure	Receptor binding affinity (IC_50_, nM)^a^				
		sst_1_	sst_2_	sst_3_	sst_4_	sst_5_
Somatostatin-14 (Viollet et al., 1995)	Ala-Gly-c[Cys-Lys-Asn-Phe-Phe-Trp-Lys-Thr-Phe-Thr-Ser-Cys]-OH	0.1–1.5	1.7	1.7	1.0–1.6	0.2–2.2
Somatostatin-28 (Viollet et al., 1995)	Ser-Ala-Asn-Ser-Asn-Pro-Ala-Met-Ala-Pro-Arg-Glu-Arg-Lys-Ala-Gly-c[Cys-Lys-Asn-Phe-Phe-Trp-Lys-Thr-Phe-Thr-Ser-Cys]-OH	0.1–4.7	0.4–5.2	0.2	0.3–1.1	0.05–0.19
Octreotide (Grace et al., 2008)	H-(D)-Phe^2^-c[Cys^3^-Phe^7^-DTrp^8^-Lys^9^-Thr^10^-Cys^14^]-Thr^15^(ol)	>1K	1.9 ± 0.3	39 ± 14	>1K	5.1 ± 1.1
ODT8-SST (Erchegyi et al., 2008)	des-AA^1,2,4,5,12,13^-(DTrp^8^)-SST	27.0 ± 3.4	41.0 ± 8.7	13.0 ± 3.2	1.8 ± 0.7	46.0 ± 27.0
Cortistatin-17 (Fukusumi et al., 1997)	Asp-Arg-Met-Pro-cyclo-[Cys-Arg-Asn-Phe-Phe-Trp-Lys-Thr-Phe-Ser-Ser-Cys]Lys	0.3–7.0	0.6–0.9	0.4–0.6	0.5–0.6	0.3–0.4
sst_1_ agonist (Erchegyi et al., 2009)	des-AA^1,4-6,10,12,13^-[DTyr^2^,D-Agl(NMe,2naphtoyl)^8^,IAmp^9^]-SST-Thr-NH_2_	0.19 ± 0.04	>1K	158.0 ± 14.0	27.0 ± 7.5	>1K
sst_2_ agonist (Grace et al., 2006)	des-AA^1,4-6,11-13^-[DPhe^2^,Aph^7^(Cbm),DTrp^8^]-Cbm-SST-Thr-NH_2_	>1K	7.5–20	942–1094	872–957	109–260
sst_2_ antagonist (Cescato et al., 2008)	des-AA^1,4-6,11-13^-[pNO_2_-Phe^2^,DCys^3^,Tyr^7^,DAph(Cbm)^8^]-SST-2Nal-NH_2_	>1K	2.6 ± 0.7	384.0 ± 97.0	>1K	>1K

* Receptor affinities were derived from competitive radioligand displacement assays in cells stably expressing the cloned human receptor using ^125^I-[Leu^8^DTrp^22^Tyr^25^]SST-28 (Grace et al., 2006, 2008; Cescato et al., 2008; Erchegyi et al., 2008, 2009) except for somatostatin-14 (Viollet et al., 1995), somatostatin-28 (Viollet et al., 1995), and cortistatin (Fukusumi et al., 1997).*

Of interest to neuroendocrinologists, somatostatin’s inhibitory action on pituitary GH release was soon extended to a wide range of hypophyseal hormones including prolactin, thyrotropin (thyroid-stimulating hormone, TSH), and adrenocorticotropic hormone (ACTH; Brown et al., 1984; Bertherat et al., 1995; Shimon et al., 1997) as well as a large number of hormones secreted from endocrine cells of the gastrointestinal tract including gastrin (Lloyd et al., 1997), cholecystokinin (CCK; Shiratori et al., 1991), secretin (Shiratori et al., 1991), gastric inhibitory peptide (GIP; Pederson et al., 1975), and neurotensin (Rokaeus, 1984), and from the pancreas glucagon (Marco et al., 1983; Strowski et al., 2000), insulin (Pederson et al., 1975; Marco et al., 1983; Strowski et al., 2000), and pancreatic polypeptide (PP; Marco et al., 1983). Studies to characterize the somatostatin receptor subtypes established that sst_2_ is primarily involved and, to a smaller extent, sst_5_ in the broad inhibitory effects of somatostatin on endocrine secretion (Lloyd et al., 1997; Strowski et al., 2000; de Heer et al., 2008). Of clinical relevance was also the compelling evidence that the majority of neuroendocrine tumors (NETs) expressed increased levels of sst_2_ leading to the development of radiolabeled peptides and peptide receptor radionuclides as diagnostic and therapeutic tools respectively for NETs (van der Hoek et al., 2010; Culler et al., 2011; Giovacchini et al., 2012; Jawiarczyk et al., 2012).

In the past decade, a significant breakthrough came from the identification of a 28-amino-acid octanoylated peptide, ghrelin in the endocrine cells of the gastric mucosa as the cognate ligand for the GH-secretagogue receptor 1a (GHS-R_1a_, later renamed ghrelin receptor, GRLN-R; Kojima et al., 2001; Davenport et al., 2005). Ghrelin’s interaction with the GRLN-R represents a third independent regulatory pathway controlling the pulsatile release of pituitary GH besides the GH-releasing hormone (GHRH) and somatostatin (Kojima et al., 2001; Malagon et al., 2003; Kineman and Luque, 2007).

Consistent with ghrelin, being predominantly produced by the endocrine cells of the stomach (Ariyasu et al., 2001), recent studies also documented that gastrointestinal NETs released ghrelin (Corbetta et al., 2003; Tsolakis et al., 2004). Therefore the regulation of ghrelin release by somatostatin will be of clinical relevance. The present review focuses on recent evidence that somatostatin signaling in the brain and the periphery exerts opposite influence on circulating ghrelin levels. The sst receptor subtype(s) involved and functional relevance of somatostatin-induced altered ghrelin plasma levels in the context of orexigenic and gastric prokinetic actions of the peptide (Kojima and Kangawa, 2005) will also be addressed.

## SOMATOSTATIN AND ITS RECEPTORS: EXPRESSION AND MODULATION OF FEEDING BEHAVIOR AND GASTROINTESTINAL MOTILITY

### EXPRESSION OF SOMATOSTATIN AND sst

Somatostatin is expressed throughout the brain except in the cerebellum (Finley et al., 1981; Johansson et al., 1984). Peptide distribution has been investigated by immunohistochemistry indicating the expression in the cortex, limbic system, central nucleus of the amygdala, sensory structures, hypothalamus (including the arcuate, ventromedial, and paraventricular nucleus), and the periaqueductal central gray (Finley et al., 1981; Johansson et al., 1984; Moga and Gray, 1985). Similarly, sst receptor mRNA expression is widely detected in the rat brain including the deep layers of the cerebral cortex, bed nucleus of the stria terminalis, basolateral amygdaloid nucleus, medial amygdaloid nucleus, paraventricular thalamic nucleus, medial preoptic nucleus, dorsomedial and ventromedial hypothalamic nucleus, arcuate and paraventricular nucleus of the hypothalamus, substantia nigra, dorsal raphe nucleus, granular layer of the cerebellum, locus coeruleus, nucleus of the solitary tract, and the dorsal motor nucleus of the vagus nerve (Fehlmann et al., 2000; Spary et al., 2008). The wide distribution of the ligand along with the receptors is consistent with the pleiotropic action of the sst signaling systems.

Somatostatin is widely expressed in the gastrointestinal tract and the pancreas, namely in endocrine mucosal D cells scattered within the stomach, small and large intestine, and in δ-cells of the pancreatic islets of Langerhans (Walsh, 1994). In addition, somatostatin is also detected in neurons of the gut enteric nervous system within both the submucosal and myenteric plexus (Costa et al., 1977). Similar to the ligand, sst receptor subtypes are widely expressed in the gastrointestinal tract. In rats, mRNA expression of sst_1_, sst_2_, sst_3_, and sst_4_ is detected in the small and large intestine without any clear predominance to one segment (Schafer and Meyerhof, 1999). The sst_1_ is the predominant form in gastrointestinal tract of mice, whereas the sst_5_ was almost undetectable (Schafer and Meyerhof, 1999). In human colon, a differential expression has been reported with sst_2_ mRNA expression on circular smooth muscle cells and sst_1-3_ mRNA on longitudinal muscle layer cells (Corleto et al., 2006). Interestingly, in the pancreas protein expression of all sst_1-5_ receptor subtypes has been identified on every major endocrine cell type, namely α, β, δ, and PP cells with a differential expression pattern between species (Ludvigsen et al., 2004).

### BRAIN ACTIONS OF SOMATOSTATIN

The first function assigned to somatostatin was the inhibition of GH release (Brazeau et al., 1973). Now somatostatin is recognized to exert several central extrapituitary actions such as the regulation of other pituitary endocrine hormones especially those responsive to stress, parasympathetic and sympathetic outflow, thermogenesis, visceral functions, and behaviors. Namely, intracerebroventricular (icv) injection of somatostatin-28 inhibited the tail suspension stress-induced rise of circulating ACTH (Brown et al., 1984). This effect was mimicked by the stable pan-somatostatin agonist, ODT8-SST but not by somatostatin-14 (Brown et al., 1984). The sst_2_ seems to play a pivotal role in these regulatory processes as sst_2_ receptor knockout mice display an increased ACTH release compared to their wild type littermates (Viollet et al., 2000).

With regard to the central actions of somatostatin to affect visceral functions, recent studies have focused on the gastrointestinal tract. ODT8-SST injected intracisternally (ic) in rats accelerated gastric emptying of a liquid non-nutrient solution. This effect is mimicked by somatostatin-28 and the sst_5_ preferring agonist, BIM-23052 but not somatostatin-14 or the agonists preferring sst_1_, CH-275, sst_2_, DC-32-87, sst_3_, BIM-23056, and sst_4_, L-803,087 (Martinez et al., 2000) indicating a prominent role of brain sst_5_ activation to induce a gastroprokinetic effect. This contention is further corroborated by the prominent mRNA expression of sst_5_ in the dorsal motor nucleus of the vagus nerve (Thoss et al., 1995). Since sst receptors are also expressed in hypothalamic brain nuclei regulating colonic functions (Mönnikes et al., 1993; Mönnikes et al., 1994; Tebbe et al., 2005) including the arcuate (sst_1-5_; Fehlmann et al., 2000; Schulz et al., 2000) and paraventricular nucleus of the hypothalamus (sst_2-4_; Fehlmann et al., 2000; Schulz et al., 2000) and the locus coeruleus (sst_2-4_; Fehlmann et al., 2000; Schulz et al., 2000), several studies investigated whether activation of central somatostatin signaling influences colonic motor functions. Stressing mice by short exposure to anesthesia vapor and icv injection of vehicle robustly stimulated propulsive colonic motor function shown by a 99% increase in fecal pellet output (Stengel et al., 2011a). This effect was completely abolished by icv pretreatment with ODT8-SST, somatostatin-28 and the selective sst_1_ agonist, whereas sst_2_ or sst_4_ agonists or octreotide (**Table 1**) had no effect. These data suggest that activation of brain sst_1_ can prevent the stress-related stimulation of colonic propulsive motor function in mice (Stengel et al., 2011a).

Central somatostatin alters food intake with a divergent action depending on the doses used: an increase is induced by icv injection of lower (picomolar) doses and a decrease following high (nanomolar) in rats (Feifel and Vaccarino, 1990). The stable pan-somatostatin agonist, ODT8-SST (Erchegyi et al., 2008) icv stimulated food intake in rats under basal as well as already stimulated conditions during the dark phase with a rapid onset (during the first hour) and a long duration of action (lasting for 4 h; Stengel et al., 2010a). ODT8-SST’s orexigenic action is sst_2_ mediated as it is reproduced by icv injection of the selective peptide sst_2_ agonist (Stengel et al., 2010d) and blocked by the selective peptide sst_2_ antagonist (Stengel et al., 2010a). This represents a central action since injected intraperitoneally at a 30-fold higher dose, the peptide did not influence food intake (Stengel et al., 2010d). The orexigenic effect of central sst_2_ stimulation observed in rats has been recently expanded to mice (Stengel et al., 2010c). A detailed analysis of the food intake microstructure using an automated food intake monitoring device showed that icv injection of the sst_2_ agonist increased the number of meals, shortened inter-meal intervals, and induced a higher rate of ingestion, whereas meal sizes were not altered (Stengel et al., 2010c). Collectively, these data indicate that brain activation of sst_2_ signaling pathways in rodents induces a rapid orexigenic response by decreasing satiety (number of meals), without influencing satiation indicated by normal meal sizes (Stengel et al., 2010c). The physiological role of brain sst_2_ signaling in modulating food intake is also supported by the decrease of nocturnal food intake induced by the peptide sst_2_ antagonist injected icv at the beginning of the dark phase (Stengel et al., 2010d). In addition, hypothalamic somatostatin shows a circadian rhythm with a peak in the early dark phase and a nadir in the early light phase (Gardi et al., 1999). Moreover, food restriction increases pituitary somatostatin release (Ishikawa et al., 1997) which could increase the drive to eat under these conditions.

### PERIPHERAL ACTIONS OF SOMATOSTATIN

Contrasting to the central effects, somatostatin’s actions in the periphery are largely inhibitory. In the stomach, somatostatin delays emptying of the food (Smedh et al., 1999) and inhibits gastric acid secretion which is mediated directly by an interaction with acid producing parietal cells but also via the reduced release of histamine from ECL cells and gastrin from G cells (Walsh, 1994). The acid-antisecretory action of somatostatin is no longer observed in sst_2_ knockout mice, indicative of a primary role of the sst_2_ (Piqueras et al., 2003). Similarly, in the small intestine somatostatin reduces intestinal peristalsis in cats, rabbits and rats, while stimulating the duodenal, jejunal, and ileal contractile response in dogs (Tansy et al., 1979). In line with the findings in rodents, somatostatin increases gastrointestinal transit time in humans (Gregersen et al., 2011). The effects on colonic motility are likely mediated by the sst_1_ and sst_2_ based on *in vitro* studies using circular and longitudinal human colonic smooth muscle cells (Corleto et al., 2006). In addition, somatostatin reduces visceral sensitivity with the sst_2_ playing a key role as indicated by visceral hypersensitivity to both mechanical and chemical stimulation in the jejunum of sst_2_ knockout mice (Rong et al., 2007). This finding is likely to be relevant in humans as well. Patients with irritable bowel syndrome injected subcutaneously with octreotide display an anti-hyperalgesic response as shown by the increased threshold of discomfort and pain using rectal barostat manometry compared to injection of placebo (Bradette et al., 1994; Schwetz et al., 2004).

## GHRELIN AND ITS RECEPTOR: EXPRESSION AND PHYSIOLOGICAL OREXIGENIC AND PROKINETIC ACTIONS

### EXPRESSION AND REGULATION OF GHRELIN AND GHRELIN RECEPTOR

Ghrelin bears a unique fatty acid (*n*-octanoyl) residue on the third amino acid which is essential for affinity and binding to the GRLN-R (Kojima et al., 1999; Kojima and Kangawa, 2005). Other dietary fatty acids of medium length can also serve as a direct source for the acylation of ghrelin (Nishi et al., 2005). The enzyme catalyzing the acylation of ghrelin was unknown for several years and just recently identified in mouse and human as a member of the membrane-bound *O*-acyltransferases (MBOATs), namely MBOAT4 which was subsequently renamed ghrelin-*O*-acyltransferase (GOAT; Gutierrez et al., 2008; Yang et al., 2008). GOAT mRNA and protein are prominently expressed in rodent and human gastric mucosa in ghrelin expressing cells (Sakata et al., 2009; Stengel et al., 2010f). In addition, GOAT protein has been detected in rodent and human intestine, pancreatic duct, gallbladder, hypothalamus and pituitary gland (Gahete et al., 2010b; Lim et al., 2011; Kang et al., 2012), rodent plasma (Stengel et al., 2010f) and human visceral and subcutaneous adipocytes (Rodriguez et al., 2012) leading to the speculation of additional acylation sites of ghrelin.

Unlike ghrelin, desacyl ghrelin, which does not bear the hydrophobic residue on the third amino acid, is the main circulating form. The acyl:desacyl ghrelin ratio is 1:3 as recently reported using an optimized blood processing protocol to improve the yield of acylated ghrelin in rats (Stengel et al., 2009). Although desacyl ghrelin does not bind to and activate the GRLN-R (Kojima et al., 1999), recent studies indicate that the peptide exerts several biological actions to influence food intake (Stengel et al., 2010e), reduce inflammatory somatic pain (Sibilia et al., 2012), muscle cachexia produced by injury in rats (Sheriff et al., 2012) and basal autophagy in human visceral adipocytes (Rodriguez et al., 2012). However, the understanding of the physiological function of this peptide is hampered by the fact that the desacyl ghrelin receptor mediating its effects is still to be identified.

Blood levels of ghrelin vary in relation with the meal pattern with an increase before meals and a decrease thereafter (Cummings et al., 2001; Tschöp et al., 2001a). In addition, fasting also increases ghrelin mRNA expression (Toshinai et al., 2001; Kim et al., 2003; Xu et al., 2009) and reduces ghrelin peptide content in the stomach (Toshinai et al., 2001; Kim et al., 2003) indicative of a stimulated production and release under conditions of food deprivation. Ghrelin levels are not only regulated by short-term variations in energy status associated with meal patterns but also by long-term changes in body weight. Ghrelin plasma levels are elevated under conditions of reduced body weight such as anorexia nervosa or tumor cachexia and reduced in obesity (Tschöp et al., 2000, 2001b; Cummings et al., 2002). Similar to the ligand, the ghrelin acylating enzyme, GOAT is regulated by the metabolic status with an increased GOAT mRNA and protein expression in rodent gastric mucosa, hypothalamus, and pituitary following a 12- or 24-h fast (Gonzalez et al., 2008; Gahete et al., 2010b; Stengel et al., 2010f). Under conditions of obesity induced by high fat diet or leptin deficiency in *ob/ob* mice, a down-regulation of GOAT mRNA occurred in the mouse pituitary, unlike the stomach or hypothalamus (Gahete et al., 2010b), while patients with obesity-associated type 2 diabetes showed higher levels of GOAT in visceral adipose tissue (Rodriguez et al., 2012). This indicates a tissue specific regulation of GOAT under conditions of obesity.

### OREXIGENIC AND PROKINETIC EFFECTS OF GHRELIN

Ghrelin is well established to stimulate food intake in line with its regulation by changes in energy status in many species including humans (Wren et al., 2000; Tang-Christensen et al., 2004; Druce et al., 2005). It is so far the only known peripherally produced and centrally acting orexigenic peptide, contrasting with the numerous anorexigenic peptides in the gut (Suzuki et al., 2011). Ghrelin’s action is blocked by pharmacological or genetic approaches using GRLN-R antagonists (Salome et al., 2009) and GRLN-R knockout mice (Sun et al., 2004; Zigman et al., 2005) indicating a key role of ghrelin-GRLN-R interaction in mediating the orexigenic response. The food intake stimulatory action can result from ghrelin crossing the blood–brain barrier and binding to GRLN-R expressed on food intake regulatory brain nuclei (Banks et al., 2002; Pan et al., 2006) or acting directly on vagal afferents which also bear the ghrelin receptor (Date et al., 2002; Sakata et al., 2003). The respective role of these pathways under nutritional changes is still to be delineated. In addition to the stimulation of food intake, ghrelin is also involved in the regulation of body weight inducing an increase of body weight following chronic infusion of the peptide. This occurs through combined actions of stimulating appetite along with increasing fat storage and reducing lipid mobilization (Tschöp et al., 2000; Strassburg et al., 2008; Davies et al., 2009). Further corroborating these findings, ghrelin and GRLN-R double knockout mice display an increased energy expenditure leading to a reduction of body weight (Pfluger et al., 2008) which, however, could not be reproduced with a single genetic deletion of either ghrelin (Sun et al., 2003; Pfluger et al., 2008) or the GRLN-R (Pfluger et al., 2008). These differential phenotypes may reflect the functional relevance of the high constitutive activity of the GRLN-R (Damian et al., 2012) and also give rise to the speculation that additional ligands for the receptor may exist (Deghenghi et al., 2001a).

## DIFFERENTIAL MODULATION OF GH RELEASE BY GHRELIN AND SOMATOSTATIN

Ghrelin exerts endocrine actions opposite to somatostatin by stimulating anterior pituitary release of GH (Kojima et al., 1999; Yamazaki et al., 2002; Kojima and Kangawa, 2011), prolactin, and ACTH (Lanfranco et al., 2010). Somatostatin’s GH inhibitory effect is mediated by the sst_2_ (Briard et al., 1997), sst_5_ (Saveanu et al., 2001) and also sst_1_ (Kreienkamp et al., 1999). The GH releasing effect of ghrelin is blunted by intravenous (iv) infusion of somatostatin in healthy volunteers (Di Vito et al., 2002) and was completely blocked in pig pituitary cells *in vitro* (Malagon et al., 2003). The GH releasing action of ghrelin is likely to not only result from inhibiting somatostatin release (Feng et al., 2011) but also from direct activation of GH release (Veldhuis et al., 2006). In addition, ghrelin and somatostatin antagonistically interact on hypothalamic arcuate cells to regulate the release of GHRH with an activation of these neurons following ghrelin and a reduction after application of somatostatin *in vitro* (Mori et al., 2010).

## ACTIVATION OF BRAIN sst_2_ SIGNALING INCREASES BASAL AND PREVENTS VISCERAL STRESS-INDUCED SUPPRESSION OF CIRCULATING GHRELIN

Based on the established centrally sst_2_-mediated orexigenic action of somatostatin (Stengel et al., 2010a,d), we further investigated whether changes in circulating ghrelin may play a role. We found that the pan-somatostatin peptide, ODT8-SST (**Table 1**) injected icv increased basal plasma acyl ghrelin levels in *ad libitum* fed rats (Stengel et al., 2010a). However, the rise was observed at 3 h postinjection and therefore unlikely to underlie the initial increase in food intake response to central ODT8-SST which occurred within the first hour. However, it may contribute to the sustained significant increase in cumulative food intake still maintained at 4 h after icv injection of ODT8-SST (Stengel et al., 2010a). Other studies showed that activation of brain sst_2_ receptor prevents the decline in circulating ghrelin induced by visceral stress. Abdominal surgery reproducibly decreased the fasting plasma levels of acyl and desacyl ghrelin with a rapid onset and long lasting effect in rats (Stengel et al., 2010b, 2011b,c). Such a response was completely prevented by the ic injection of ODT8-SST as monitored 50 min postsurgery at a time where the peptide induces a 31 and 46% rise in fasting circulating acyl and desacyl ghrelin, respectively in sham animals (Stengel et al., 2011b). In addition, the sst_2_ selective peptide agonist injected ic also prevented the postoperative decline in plasma levels of acyl ghrelin, whereas sst_1_ and sst_4_ agonists (**Table 1**) did not when tested under the same conditions (Stengel et al., 2011b).

Of interest was the demonstration that ic ODT8-SST or sst_2_ agonist in addition to preventing the decline in acyl ghrelin induced by abdominal surgery also blunted the postoperative suppression of food intake (Stengel et al., 2011b). However, the observed prevention of declining circulating ghrelin levels is not the main factor for the restoration of the orexigenic response postsurgically. The peripheral blockade of the GRLN-R using the GRLN-R antagonist ([D-Lys^3^]-GHRP-6) injected intraperitoneally did not modify the food intake stimulating effect of ic ODT8-SST (Stengel et al., 2011b). ODT8-SST injected icv also restored gastric emptying inhibited by abdominal surgery to levels observed under basal conditions, an effect mimicked by the sst_5_ but not the sst_1_, sst_2_, and sst_4_ agonists in rats (Stengel et al., 2011b). Similar to the effect on food intake, this action was not mediated by acyl ghrelin as the intraperitoneal injection of the GRLN-R antagonist did not influence the gastroprokinetic action of ODT8-SST postsurgery (Stengel et al., 2011b).

Taken together, these data indicate that the central activation of sst_2_ increases basal or prevents the surgical inhibition of circulating levels of ghrelin and food intake while activation of sst_5_ restores postoperative gastric ileus in rats. It also points to a differential role of brain sst subtypes 2 and 5 in preventing the stress-related suppression of ghrelin release/food intake and gastric emptying, respectively. In addition, the restoration of suppressed circulating acyl ghrelin levels after surgery does not play a major role as underlying mechanisms through which ODT8-SST injected into the brain exerts its prokinetic and orexigenic effects. Other studies showed that activation of sst_2_ and sst_5_ receptors in the brain inhibits stimulated CRF release in the hypothalamus and acute stress-related CRF mediated ACTH release (Brown et al., 1984; Tizabi and Calogero, 1992; Saegusa et al., 2011; Tringali et al., 2012). Brain CRF acting on CRF receptors is involved in the stress-related decrease in feeding behavior, ghrelin secretion, and gastric emptying (Hotta et al., 1999; Sekino et al., 2004; Taché and Bonaz, 2007; Yakabi et al., 2011). Therefore, it may be speculated that central activation of sst_2_ and/or sst_5_ dampens hypothalamic CRF activated by abdominal surgery (Wang et al., 2011) which may have a bearing with preventing the reduction in feeding, gastric emptying, and circulating ghrelin induced by abdominal surgery (Stengel et al., 2011b).

## ACTIVATION OF PERIPHERAL sst_2_ SIGNALING INHIBITS CIRCULATING GHRELIN

In contrast to the rise in circulating ghrelin induced by central administration of somatostatin agonists, convergent *in vivo* and *in vitro* rodent studies established that somatostatin or the stable agonists octreotide and SOM230 act peripherally through the sst_2_ to reduce ghrelin release (Seoane et al., 2007; Iwakura et al., 2010; Lu et al., 2012) resulting in lower circulating levels (Shimada et al., 2003; Silva et al., 2005; de la Cour et al., 2007). Such a response is in line with the inhibitory effects of somatostatin-sst_2_ on the endocrine secretion of other intestinal hormones (Pederson et al., 1975; Marco et al., 1983; Rokaeus, 1984; Shiratori et al., 1991; Strowski et al., 2000). Likewise, in humans, peripherally injected somatostatin (Broglio et al., 2002; Norrelund et al., 2002) and somatostatin agonists such as octreotide (Barkan et al., 2003) reduce circulating ghrelin in healthy subjects. Of relevance, chronic subcutaneous infusion of the sst_2_/sst_3_/sst_5_ agonist octreotide-induced suppression of ghrelin plasma levels is not subject to rapid desensitization in rats and is likely to be sst_2_ mediated based on the prominent sst_2_ mRNA expression in the rat stomach (Silva et al., 2005). Further support for a role of sst_2_ came from immunofluorescent double labeling studies detecting the protein expression of sst_2_ on ghrelin-producing X/A-like cells of the rat stomach (Stengel et al., 2011c) and similarly on human ghrelin-producing gastric mucosal P/D_1_ cells (Fischer et al., 2008). Moreover, the selective peptide sst_2_ agonist (**Table 1**) injected intravenously decreased circulating levels of acyl and desacyl ghrelin with a rapid onset (0.5 h) and a long duration of action (still visible at 2 h; Stengel et al., 2011c). Lastly, the selective peptide sst_2_ antagonist (**Table 1**) injected intravenously prevents the decline in circulating ghrelin induced by the stimulation of endogenous peripheral somatostatin in rats (Stengel et al., 2011c).

The physiological role of peripheral somatostatin-sst_2_ signaling in the regulation of ghrelin was investigated under conditions of stimulation of endogenous gastric somatostatin. Urethane is well established to stimulate gastric somatostatin mRNA expression and peptide release in rats (Yang et al., 1990). Under these conditions, plasma ghrelin levels are decreased and the selective peptide sst_2_ antagonist (**Table 1**) injected intravenously prevents the decline in circulating ghrelin induced by urethane (Stengel et al., 2011c). Convergent reports showed that abdominal surgery induces a rapid and sustained inhibition of circulating levels of ghrelin in rats (Stengel et al., 2010b, 2011b,c). Likewise, iv injection of the selective peptide sst_2_ antagonist (**Table 1**) blocked the abdominal surgery-induced decrease of plasma ghrelin at 0.5 h postsurgery (Stengel et al., 2011c). The peptide is likely to act through paracrine transmission since somatostatin positive D cells directly contact ghrelin immunoreactive X/A-like cells in the rat stomach (Shimada et al., 2003). Interestingly, following abdominal surgery, acyl ghrelin was reduced more rapidly compared to desacyl ghrelin which was associated with a reduction of gastric as well as plasma concentrations of GOAT (Stengel et al., 2011c). Since blockade of peripheral sst_2_ signaling restores circulating levels of ghrelin (Stengel et al., 2011c), these data collectively suggest that peripheral somatostatin may blunt gastric GOAT mRNA expression and thereby negatively affect the acylation of ghrelin. In primary pituitary cell cultures somatostatin was reported to reduce GOAT mRNA expression and somatostatin knockout mice showed higher GOAT mRNA expression in the pituitary gland than the wild type (Gahete et al., 2010b). Based on these findings, somatostatin may influence ghrelin signaling not only *via* a direct inhibition of secretion but also by modulating the ghrelin activating enzyme GOAT. Further support for a physiological inhibitory action of peripheral somatostatin on ghrelin signaling came from somatostatin knockout mice that displayed an increased gastric ghrelin expression and higher circulating ghrelin levels compared to their wild type littermates (Luque et al., 2006a). These data indicate that endogenous somatostatin exerts a physiological inhibitory tone on gastric ghrelin synthesis and release.

During the past years, several clinical studies described ghrelin-producing NETs (Papotti et al., 2001; Volante et al., 2002; Taal and Visser, 2004) including six insulinomas, gastrinomas, vasoactive intestinal polypeptide (VIP)omas, non-functioning tumors (Volante et al., 2002) and as part of the multiple endocrine neoplasia type 1 (MEN-1; Iwakura et al., 2002; Raffel et al., 2005; Ekeblad et al., 2007). Moreover, ghrelinomas have been identified to originate from the stomach or pancreas and were associated with very high ghrelin levels (Corbetta et al., 2003; Tsolakis et al., 2004). Growing evidence from clinical reports indicates that in addition to the surgical, chemotherapeutic, alpha-interferon, and local radiation treatments, the use of somatostatin or somatostatin analog is an established treatment option for gastroenteropancreatic NETs (Pavel and Wiedenmann, 2011). Based on the evidence described above, the determination of the sst subtype expressed on those ghrelinoma tumor cells followed by the use of peripherally acting selective sst compounds may result in an even more targeted approach.

## INTERACTION OF CORTISTATIN WITH GHRELIN SIGNALING

In 1996, the discovery of a new peptide sharing 11 of its 14 amino acids with somatostatin-14 was named cortistatin based on its predominant cortical expression and ability to depress cortical activity (de Lecea et al., 1996). Despite the chemical structure homology with somatostatin, both peptides are derived from distinct genes (de Lecea et al., 1997b). Similar to pro-somatostatin, processing of cortistatin precursor generated two mature products, cortistatin-14 and -29 in rodents and cortistatin-17 and -29 in humans (Fukusumi et al., 1997; Spier and de Lecea, 2000). Cortistatin is widely expressed in the brain, namely in the cortex and hippocampus, and although regional overlap exists, the distribution pattern differs from that of somatostatin (de Lecea et al., 1997a). Likewise, the peripheral expression pattern of cortistatin (e.g., in adrenal, thyroid, and parathyroid gland, testis, pancreas, kidney, lung, liver, stomach, ileum, jejunum, colon, endothelial, and immune cells) does not fully match that of somatostatin (Papotti et al., 2003; Dalm et al., 2004; Xidakis et al., 2007). Consistent with being a close somatostatin endogenous analog, cortistatin contains the FWKT tetramer crucial for sst binding, and therefore displays high-affinity (1–2 nM) to all five sst subtypes where the peptide acts as an agonist (Fukusumi et al., 1997; Siehler et al., 2008). However, emerging evidence indicates that cortistatin induces distinct central and peripheral effects that differ from those exerted by the somatostatin-sst interaction such as central acetylcholine release, reduction of locomotor activity, depression of cortical activity, induction of slow wave sleep, anti-inflammatory and immunomodulatory effects, and reduction of vascular calcium deposition (for review, see Spier and de Lecea, 2000; Broglio et al., 2007; Gonzalez-Rey and Delgado, 2008).

The existence of a specific cortistatin receptor has not been identified yet but differential actions between somatostatin and cortistatin may possibly reside in the ability of cortistatin to bind and to activate the GRLN-R, whereas somatostatin does not (Deghenghi et al., 2001b; Muccioli et al., 2001). Divergent from native somatostatin, however, synthetic somatostatin agonists, lanreotide, octreotide, and vapreotide bind to the GRLN-R in human pituitary tissue (Deghenghi et al., 2001c) in addition to their selective affinity for sst_2_ > sst_5_ > sst_3_ (Bauer et al., 1982; Reichlin, 1983; Redding and Schally, 1984). In addition to binding studies, few functional findings also support the possibility that cortistatin may be another endogenous high-affinity ligand of the GRLN-R. Cortistatin has been reported to inhibit vascular calcification induced experimentally in rats through activation of the GRLN-R receptor rather than sst or Mrg X2 (Liu et al., 2010). Of interest was the demonstration that cortistatin selectively upregulates the GRLN-R mRNA expression in cultured rat vascular smooth muscle cells, further indicative of an interaction between cortistatin and ghrelin signaling (Liu et al., 2010). Other studies in primates and mice demonstrated that endogenous cortistatin, unlike somatostatin, is involved in the stimulation of pituitary prolactin release, an effect that is blocked *in vitro* by the GRLN-R antagonist (Cordoba-Chacon et al., 2011). However, most of the endocrine studies performed *in vivo* or *in vitro* showed parallel inhibitory responses between cortistatin and somatostatin consistent with the activation of classical sst subtypes (Broglio et al., 2008) with some exceptions (Prodam et al., 2008). This is further corroborated by the finding that cortistatin knockout mice display elevated circulating acyl ghrelin levels associated with an upregulated gastric ghrelin and GOAT expression (Cordoba-Chacon et al., 2011) indicating an inhibitory tone of endogenous cortistatin on ghrelin signaling.

To further delineate the actions of cortistatin mediated *via* the GRLN-R another peptide, cortistatin-8, has been shown to bind to the GRLN-R while being devoid of affinity to the sst subtypes (Luque et al., 2006b). However, in one clinical study cortistatin-8 did not influence spontaneous pituitary hormone secretion (GH, prolactin, and ACTH) and did not interfere with ghrelin’s endocrine responses when given in equimolar dose ratios in healthy human subjects (Prodam et al., 2008). Therefore, additional specific tools may be needed to characterize a possible direct link between the ghrelin and somatostatin signaling system *via* interaction on the GRLN-R.

## SUMMARY

In summary, somatostatin robustly affects circulating levels of ghrelin through interaction with the sst_2_. However, alterations vary with the site of action. Central somatostatin elevates plasma levels of acyl and desacyl ghrelin *via* interaction with brain sst_2_ and counteracts the visceral stress-related decrease in circulating ghrelin through pathways still to be elucidated in rodents. By contrast, the activation of peripheral somatostatin-sst_2_ inhibits circulating ghrelin levels in experimental and clinical studies and mediates the decline in circulating ghrelin induced by abdominal surgery in rodents likely *via* a paracrine action of somatostatin on sst_2_-bearing ghrelin cells in the stomach. Of interest, cortistatin, the other member of the somatostatin family, in addition to binding to sst_1-5_, also binds to and activates the GRLN-R. There is evidence that the peptide can exert a dual influence on ghrelin, by inhibiting its release through interaction with sst_2_ located on gastric ghrelin cells while activating GRLN-R at ghrelin’s tissue targets.

## Conflict of Interest Statement

The authors declare that the research was conducted in the absence of any commercial or financial relationships that could be construed as a potential conflict of interest.
